# A Case of Posthumous Variola

**Published:** 1841-04

**Authors:** 


*A Case of Posthumous Variola.* By Dr. Chansarel.—A girl, aged 5 years and 8 months, who had been vaccinated, was suddenly attacked, when in perfect health, with a varioloid eruption and fever. About twelve hours afterwards, the redness of the skin and the pimples totally disappeared, cerebral phenomena supervened, and death twenty-six hours after the first appearance of illness. On the next day, thirteen hours after death, "I saw," says M. Chansarel, "to my great astonishment, a great number of pimples which had appeared after death, and which were much more developed than those I had seen during the life of the child. They were seated on the face, neck, chest, and buttocks; those in the latter region being larger, equalling the size of a lentil. They had the aspect of variolous pustules, surrounded by a red areola, depressed in their centre, and contained a small quantity of fluid. The body exhaled a fetid odour, and exhibited some red and livid spots, putrefaction not having been so slow as it appeared."—*Ibid., from Bulletin Medical du Midi. Juin, Juillet,* 1840. *Ibid.*

